# The mechanism of spin-phonon relaxation in endohedral metallofullerene single molecule magnets†

**DOI:** 10.1039/d4sc07786e

**Published:** 2025-06-09

**Authors:** Tanu Sharma, Rupesh Kumar Tiwari, Sourav Dey, Lorenzo A. Mariano, Alessandro Lunghi, Gopalan Rajaraman

**Affiliations:** a Department of Chemistry, Indian Institute of Technology Bombay Mumbai Maharashtra 400076 India rajaraman@chem.iitb.ac.in; b School of Physics and AMBER Research Centre, Trinity College Dublin 2 Ireland lunghia@tcd.ie

## Abstract

This study presents the investigation of spin-phonon coupling mechanisms in fullerene-based single-molecule magnets (SMMs) using *ab initio* CASSCF combined with DFT calculations. While lanthanide-based SMMs, particularly those with Dy^III^ ions, are known for their impressive blocking temperatures and relaxation barriers, endohedral metallofullerene (EMFs) offer a unique platform for housing low-coordinated lanthanides within rigid carbon cages. We have explored the spin dynamics in DyScS@C_82_ exhibiting among the highest blocking temperature (*T*_B_) reported. Through our computational analysis, we reveal that while the fullerene cage enhances crystal field splitting and provides structural stability without significantly contributing to spin-relaxation driving low-energy phonons, the internal ionic motion emerges as the primary factor controlling spin relaxation and limiting blocking temperature. This computational investigation into the spin dynamics of EMF-based SMMs provides key insights into their magnetic behaviour and suggests potential strategies for improving their performance towards futuristic SMMs.

## Abstract 2

Low-lying phonons associated with the specific bending motion of cluster atoms with high spin-phonon coupling play a crucial role in the system's relaxation dynamics.

## Graphical abstract

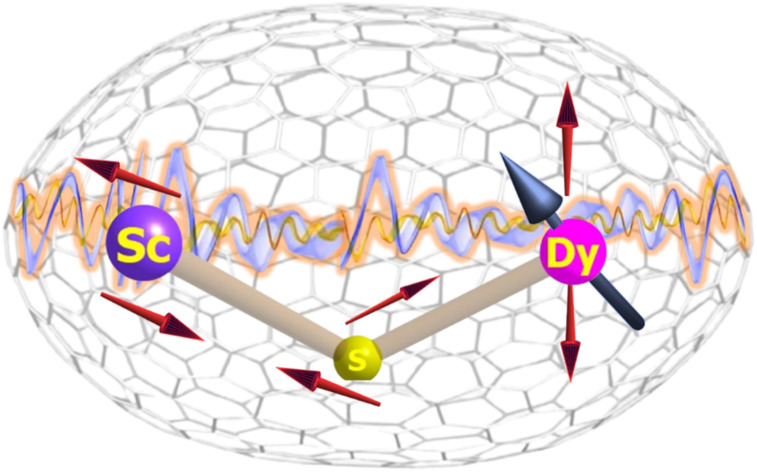

## Introduction

Due to their very high blocking temperatures and large thermal barriers to relaxation, Dy^III^-based single-molecule magnets (SMMs) are of great interest.^1–6^ Among many challenges in taking these molecules to end-user applications, enhancing the barrier height for magnetization reversal (*U*_eff_) and blocking temperature below which the magnetisation is fully frozen (*T*_B_) are considered crucial. Coupling magnetic lanthanide ions to strong axial ligand fields is one of the most promising approaches to producing high-performing SMMs.^1,3^ This has produced SMMs with a barrier height of magnetisation reversal as large as 1500 cm^−1^, achieving one of the aforementioned goals.^1,2^ Although such large barriers have been achieved so far the *T*_B_ values remain modest at 80 K.^1^ While theoretical advances have propelled the understanding of *U*_eff_ through various intuitive ligand design principles that have shaped the field of lanthanide SMMs over the past few decades, the comprehension of *T*_B_, the most critical factor governing magnetic performance, remains largely unclear. This is evident from the fact that many molecules with high *U*_eff_ display low *T*_B_, and *vice versa*, underscoring the need for a molecular-level understanding beyond simple electron density considerations (such as prolate *versus* oblate shapes). To achieve the next breakthrough in enhancing *T*_B_ values, it is essential to focus on this deeper molecular understanding. The *T*_B_ values and the corresponding relaxation times are strongly linked to the spin-phonon relaxation mechanism, an area of significant recent research aimed at providing insights to improve *T*_B_.^1,2,7–9^

The mechanism of spin-phonon relaxation has been explored so far in a handful of examples offering some guidelines to enhance the *T*_B_ values.^8,10,11^ These guidelines include: (i) achieving strong crystal-field splitting of the Kramers doublets,^12,13^ (ii) ensuring that the crystal field splitting exhibits minimal transverse anisotropy, which drives quantum tunnelling of magnetization (QTM),^14,15^ (iii) reducing the vibrational density of states at resonant frequencies,^14^ (iv) minimizing low-energy vibrations,^16^ (v) utilizing rigid ligands that can isolate intramolecular motions from low-energy acoustic vibrations,^7,17^ and (vi) employing ligands with donor atoms whose local charge remains stable despite local vibrations.^8^ Controlling these factors is very challenging, as crystal field parameters and reducing transverse anisotropy are correlated to the geometry and local point group of the molecules. Strong crystal field splitting and less transverse anisotropy can be achieved with low-coordinated lanthanides or with higher oxidation state,^18^ but synthesizing such molecules is challenging, and even if they are synthesised with very bulky ligands, they often encounter other weak non-covalent interactions such as Ln⋯H–C agostic interactions that facilitate transverse anisotropy as demonstrated in several cases.^2,19,20^ Moreover, traditional organometallic complexes often have ligands with loosely bound atoms, like hydrogen, whose vibrations also contribute to spin-phonon relaxation.^2^ Synthesis of the rigid lanthanide low-coordinate molecule without atoms such as –H are extremely challenging and has not yet been achieved.

However, in the endohedral metallofullerenes (EMFs) class of lanthanide-based SMMs, these conditions can be met easily.^21–26^ The EMF-based SMMs feature rigid cages that can stabilize low-coordinated lanthanides inside their cage structure.^26,27^ These compounds contain atoms or small clusters of atoms encapsulated by fullerenes, where cage shields the magnetic ion from the decoherence caused by external noise and hence stabilize atomic configurations that are not achievable in conventional molecules. These unique circumstances can produce very effective, precisely controlled SMMs. Although not all conditions are met, this type of molecule often satisfies conditions (i), (ii), (iv) (v) and (vi). This is demonstrated by the fact that lanthanide EMFs are reported as high-performing SMMs far more frequently than any other class of molecules (see Table S1†). Despite these key advantages, the *T*_B_ that has been reached with this class of molecule is relatively small compared to the best-in-class dysprosocenium class of molecules. This is linked to the spin-phonon relaxation mechanism, but a clear understanding of this process for this class of SMMs is still missing. Investigating the spin-phonon relaxation mechanism in these fullerene molecules could provide valuable insights, potentially leading to methods for suppressing or minimizing this effect.

Endohedral metallofullerenes can be broadly classified into two main categories. The first category consists of traditional metallofullerenes, where only metal atoms are encapsulated within the fullerene structure. In contrast, the second category, known as cluster fullerenes, encloses not only metal atoms but also non-metals such as nitrides, oxides, sulphides, carbides, and related cyanides within the fullerene shell.^22,28–37^ Although among all fullerene based SMMs, Dy@C_s_(6)-C_81_N possess the largest *T*_B_ value,^25^ among cluster fullerenes with single magnetic centre, DyScS@C_82_ exhibit the one of the best *T*_B_ value of 7.3 K. Almost all reported EMFs exhibiting SMM characteristics within this range, suggesting a common spin-phonon relaxation mechanism in this class of molecules that may limit the achievement of higher *T*_B_ values. In this manuscript, we investigated the spin-phonon relaxation processes in one such cluster fullerene DyScS@C_82_ using state-of-the-art *ab initio* spin relaxation simulations. To the best of our knowledge, our study marks the first implementation of *ab initio* spin-phonon relaxation calculations in the context of fullerene-based SMMs. Our goal is to (i) elucidate the mechanism of spin-phonon relaxation, (ii) compute relaxation times for comparison with experimental data, (iii) analyse the vibrational modes responsible for spin-phonon relaxation, and (iv) provide general guidance on how to achieve larger *T*_B_ values in this class of molecules.

In the original paper by Echegoyen and co-workers,^37^ the synthesis and characterisation of two new dysprosium-containing mixed-metallic sulphide cluster fullerenes, DyScS@C_s_(6)–C_82_ and DyScS@C_3v_(8)−C_82_ were reported. These compounds were isolated and analysed using various techniques, including mass spectrometry, Vis-NIR spectroscopy, cyclic voltammetry, and single-crystal X-ray diffraction. For this manuscript, we chose DyScS@Cs(6)−C_82_ for our calculations due to its higher *U*_eff_ barrier and longer relaxation times in the absence of external magnetic fields compared to DyScS@C_3v_(8)−C_82_. In the remainder of the manuscript, we will refer to DyScS@C_s_(6)-C_82_ simply as DyScS@C_82_.

## Computational details

To execute geometry optimizations, as well as simulations of Γ-point phonons, the CP2k program package^38–40^ was used. The CP2k software uses a hybrid basis set technique called the Gaussian and Plane Wave Method (GPW),^39,40^ in which the electronic charge density is represented by an auxiliary plane-wave basis set and Kohn–Sham orbitals are enlarged using contracted Gaussian-type orbitals (GTOs). A large cell of 20 Å^3^ containing a single fullerene unit was used for geometry optimization in absence of periodic boundary conditions. Utilising their relativistic norm-conserving pseudopotentials (Goedecker, Teter, and Hutter)^41^ optimised for the PBE functional.^42^ The Dy^III^ ion was replaced by the Y^III^ ion to avoid convergence issues with the DFT framework, however the atomic mass of Dy was used in order to create the vibrational modes. The DZVP-MOLOPT-GTH basis set (valence double-zeta (*ζ*) plus polarisation, molecularly optimised, Goedecker–Teter–Hutter) for all atoms (H, C) and the DZVP-MOLOPT-SR-GTH basis set (valence double-zeta (*ζ*) plus polarisation, molecularly optimised, short range Goedecker–Teter–Hutter) for Y, as implemented in the CP2k, were used for all calculations.^40,43^ Furthermore, 1000 Ry was used as the energy cut-off for the plane wave basis set. For geometry optimization, a very tight force convergence criterion of 10^−8^ au and an SCF convergence criterion of 10^−8^ au for energy were employed (see input file in ESI†). Here it is important to discuss that the EMFs are known to exhibit a significant degree of structural disorder due to the dynamic nature of the encapsulated metal cluster and the flexibility of the fullerene cage. In our study, we have initiated the modeling from an optimized geometry to provide a well-defined and reproducible starting point for theoretical analysis. While this approach offers clarity and computational tractability, it does not fully capture the inherent disorder present in experimental systems. To better address these complexities, molecular dynamics (MD) simulations can be employed to explore the range of accessible conformations and mimic dynamic disorder.^5^ Such techniques although beyond the scope of this paper, offer a promising route to understand how structural fluctuations influence the magnetic properties of EMF-based single-molecule magnets.

The crystal field parameters for the geometries were computed using the ORCA 5.0 suite of software.^44^ For the Dy^III^ ions, magnetic properties were derived from CASSCF calculations with an active space of seven 4f orbitals containing nine electrons (9,7), considering all solutions with multiplicities of 6. The basis sets DKH-SVP for C atoms, and SARC-DKH-TZVP for Dy atoms,^45^ DKH-TZVP for Sc and S atoms were utilized.^46,47^ Also, we have performed CASSCF/RASSI-SO^48^/SINGLE_ANISO calculations using the Molcas 8.2 package in order to compute the static relaxation mechanism in DyScS@C_82_ molecule and the {Dy–S–Sc}^4+^ fragment.^49,50^ We have employed the ANO-RCC-TZVP basis set^51^ for Dy^III^ ions and ANO-RCC-DZVP for rest of the atoms. We have used 21 sextets in CASSCF and RASSI-SO calculations as demonstrated earlier in this class of compounds.^5,52,53^ We have also used the Gaussian 16 package^54^ for the Atoms in Molecules (AIM) analysis, employing the UB3LYP functional^55–57^ with the SDD basis set^58^ for Y, CSDZ^59,60^ for Dy^III^, and 6-31G* for the remaining atoms.^61^ Given the heavy computational requirements of spin-phonon calculations, due to the need to sample many geometries and electronic states, dynamical correlation was excluded from our study. However, previous investigations^18,52,62^ support that this approximation maintains qualitative consistency in magnetic trends.

### Calculations of the crystal field and spin-phonon coupling coefficients

The effective crystal-field Hamiltonian employed here correspond to1
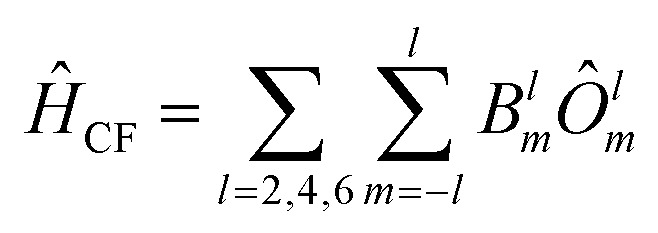


The parameters *B*^*l*^_*m*_are the CF Hamiltonian coefficients, and the operators *Ô*^*l*^_*m*_ are tesseral functions of the total angular momentum operator *J*, with rank l and order m.^63^ The lowest 2*J*+1 *ab initio* wave functions are matched one-to-one with the magnetic ground state of the ion to determine the CF Hamiltonian coefficients from first principles. To achieve this, the spin Hamiltonian |*J̃m*_*J*_〉 is obtained by diagonalizing the operator *Ĵ*_z_ within the basis of the lowest 2*J*+1 *ab initio* wave functions.^64^ Subsequently, the *ab initio* energy matrix is represented in this basis, and the parameters in eqn (1) are adjusted to reproduce the *ab initio* energy matrix elements.^64^ These values enable the calculation of the magnetic anisotropy tensor across all temperatures and the magnetization in every spatial direction. The crystal field Hamiltonian, as described in eqn (1), represents the energy levels of the ground-state electronic multiplet for the equilibrium geometry of the molecule. However, due to thermal energy, the molecular geometry is constantly fluctuating. These fluctuations lead to the modulation of spin properties, namely spin-phonon coupling, causing transitions between different electronic states until a thermal equilibrium is achieved between the electronic states and the lattice. Therefore, it is necessary to describe the spin, the phonons, and their interaction in detail in order to provide a quantum mechanical explanation of spin-dynamics. To derive the spin-phonon coupling coefficients (∂*Ĥ*_s_/∂*Q*_α_), *B*^*l*^_*m*_ that occur in eqn (1) are numerically differentiated with respect to the atomic displacements described by *Q*_α_.2
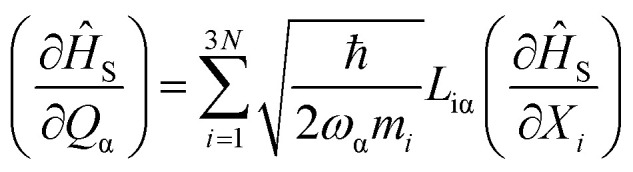
where *N* is the number of atoms in the unit cell, *Q*_α_ is the displacement vector connected to the α-phonon, and *L*_*i*α_ and *ω*_α_ are the eigenvectors of the Hessian matrix and the angular frequency of the phonon, respectively. By using numerical differentiation, the spin Hamiltonian's first-order derivatives (∂*ĥ*_S_/∂*X*_*i*_) with respect to the Cartesian degree of freedom *X*_*i*_ are determined. Four samples are taken between ±0.1 Å for each molecular degree of freedom.

### Calculation of spin-relaxation

The total Hamiltonian operator is defined as:

3 Ĥ = *Ĥ*_S_ + *Ĥ*_Ph_ + *Ĥ*_S−Ph_

where the spin and phonon Hamiltonians are represented by the first two terms, respectively, and the coupling between these subsystems is represented by the third term. All-electron system's low-lying electronic states are described by the spin Hamiltonian. A straightforward sum of harmonic oscillators is used to simulate the phonon Hamiltonian. It is worth noting that while incorporating anharmonic interactions would provide a more complete picture, doing so requires computing linewidths from first-principles methods and perturbation theory— an approach that remains computationally expensive and not necessarily more accurate.^8,65–68^

After obtaining the eigenstates, |*a*〉, and eigenvalues, *E*_a_, of these operators, spin dynamics can be simulated by calculating the *W*_ab_, or transition rate between distinct spin states.^69^ In molecular Kramers systems with high magnetic anisotropy, one- and two-phonon processes contribute to spin relaxation. When one-phonon processes are taken into account, the transition rate between spin states, *Ŵ*^1−Ph^_ba_ is4
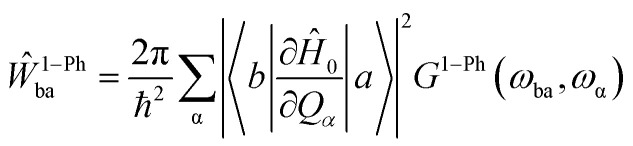
where the phrase (∂*Ĥ*_0_/∂*Q*_α_) indicates the strength of the coupling between spin and the α-phonon *Q*_α_, and ℏ*ω*_ba_ = *E*_b_ – *E*_a_. The *G*^1−Ph^ function reads

5 *G*^1−Ph^(*ω*,*ω*_α_) = *δ*(*ω* − *ω*_α_)*n̄*_α_ + *δ*(*ω* + *ω*_α_)(*n̄*_α_+ 1)

where the Bose–Einstein distribution accounting for the thermal population of phonons is represented by 
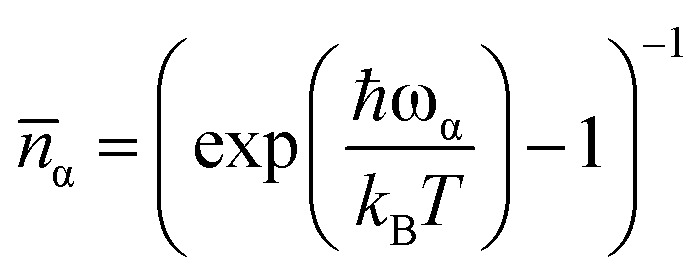
, the Boltzmann constant is represented by *k*_B_, and the Dirac *δ* functions enforce energy conservation during the absorption and emission of phonon by the spin system, respectively, here *δ* functions has been approximated using Gaussian smearing. The finite differentiation is used to calculate lattice harmonic frequencies (*ω*_α_/2π) and normal modes (*Q*_α_) following geometry optimisation using DFT. The Orbach relaxation mechanism is explained by eqn (2), where the spin moves from the fully polarised state *M*_J_ = *J* to an excited state with an intermediate value of *M*_J_ before it can emit phonons again and return to *M*_J_ = −*J*. This process also occurs for states represented by the total angular momentum, *J*. A further route of relaxation to equilibrium is offered by two-phonon processes, leading to the Raman mechanism. Two-phonon spin-phonon transitions, or *Ŵ*^2−Ph^_ba_are modelled as6
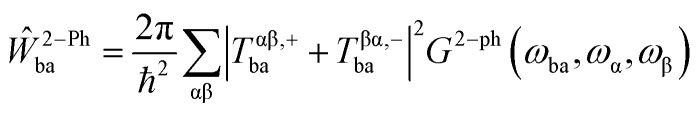
where *T*^αβ,±^_ba_ is7
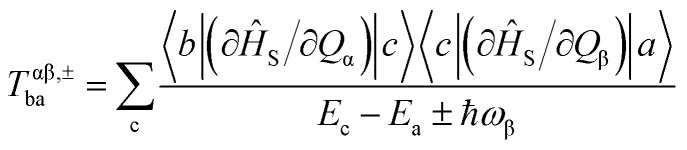
*T*^αβ,±^_ba_ entails the simultaneous contribution of all spin states |*c*〉, often known as a virtual state. All two-phonon processes, such as the absorption of two phonons, the emission of two phonons, or the absorption of one phonon and the emission of a second, are taken into account by *G*^2−Ph^. The latter method, which in this instance yields a *G*^2−Ph^ reading, determines the Raman relaxation rate.

8 *G*^2−Ph^ (*ω*,*ω*_α_,*ω*_β_) = *δ*(ω − *ω*_α_ + *ω*_β_) *n̄*_α_(*n̄*_β_ + 1)

After computing each matrix element *Ŵ*^*n*−Ph^_ba_, *τ*^−1^ can be anticipated by diagonalizing *Ŵ*^*n*−Ph^_ba_ and determining the smallest nonzero eigenvalue. The Orbach contribution to the relaxation rate, *τ*^−1^_Orbach_, is obtained from the analysis of *Ŵ*^1−Ph^, while the Raman contribution, *τ*^−1^_Raman_, is obtained from *Ŵ*^2−Ph^. Hence, the *Ŵ* calculation of the entire relaxation time is *τ*^−1^ = *τ*^−1^_Orbach_ + *τ*^−1^_Raman_. For Raman, we have rotated the molecule in the Eigen frame of g-tensors of the ground state KD and then applied a small magnetic field to lift the degeneracy.

## Results and discussion

### Structure, bonding and the static relaxation mechanism in DyScS@C_82_

**Fig. 1:**
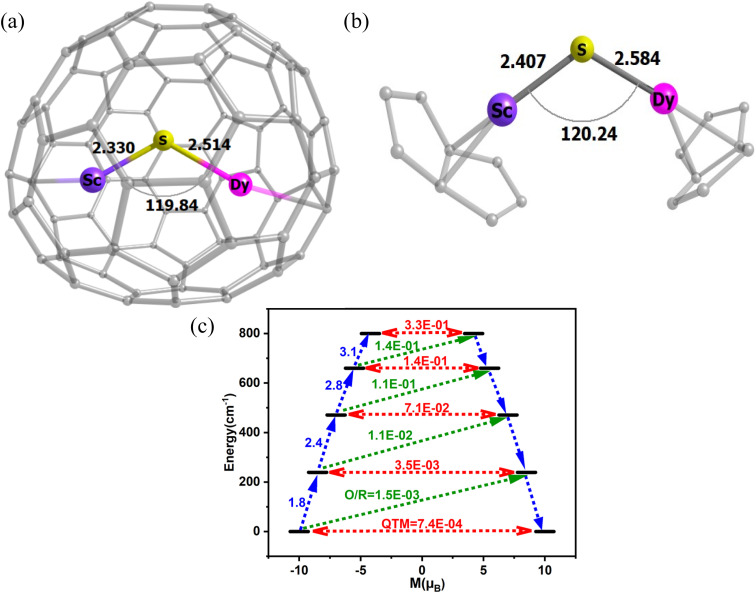
(a) DFT optimised structure of DyScS@C_82_, (b) DFT optimised structure of DyScS(C_8_H_6_)_2_, (c) *ab initio* computed magnetic blockade diagram in DyScS@C_82_. The thick black line indicates the KDs as a function of the computed magnetic moment. The green/blue arrows show the possible pathway through Orbach/Raman relaxation. The dotted red lines represent the presence of QTM/TA-QTM between the connecting pairs. The numbers provided at each arrow are the mean absolute values for the corresponding matrix element of the transition magnetic moment. Colour code: Dy^III^ -pink, S-yellow, C-grey and Sc-violet. Bond lengths and bond angle are given in Å and °, respectively.

To start, we optimized the geometry of DyScS@C_82_ using DFT methods (see Computational Details). The optimized structure reveals a Dy–S bond distance of 2.514 Å (Fig. 1(a)), which falls within the range reported for the X-ray structure, despite its high degree of disorder, with a Dy–S–Sc angle of 119.8°. Additionally, we optimized the pentalene clusters DyScS(C_8_H_6_)_2_ to assess the influence of the cage on the structure and magnetic properties.^70^ In DyScS(C_8_H_6_)_2_, the Dy–S bond distances is 2.584 Å, with a Dy–S–Sc angle of 120.4°, closely matching the value observed in the cage (Fig. 1(b)).

To further investigate bonding characteristics, we performed Atoms in Molecules (AIM) analysis within the DFT framework (see Table S2† for details). In both DyScS@C_82_ and DyScS(C_8_H_6_)_2_, the Dy^III^–cage and Dy^III^–S interactions exhibit a |*V*(r)/*G*(r)| ratio of 1.15–1.28, suggesting a bonding nature that lies between covalent and ionic. Additionally, the positive Laplacian of the electron density (∇^2^*ρ*(r) > 0) suggests ionic interactions, whereas the negative total energy density (*H*(r) < 0) implies covalent character.^71^ Consequently, these interactions can be classified as polar covalent. To further evaluate metal–cage interactions, we calculated the delocalization index *δ*(Dy, C_cage_), which falls within the range of 0.101–0.345. This is consistent with typical metal–cage interactions observed in EMFs (Fig. S1 and Table S3†).^72^

Furthermore, we performed SA-CASSCF/RASSI/SINGLE_ANISO calculations using MOLCAS to evaluate the magnetic properties of DyScS@C_82_ and DyScS(C_8_H_6_)_2_. For DySc@C_82_, the splitting of eight Kramers doublets (KDs) is found to be 1011.5 cm^−1^. The *g*_xx_, *g*_yy_, and *g*_zz_ values of the ground KD are 0.002, 0.003, and 19.925 (Table S4†), respectively, indicating strong Ising anisotropy. The angle between the ground KD g_zz_ and the excited state g_zz_ remains small up to the second excited KD (2.5–5.9°) but increases to 20.9° in the third excited KD (Table S4†).

The third excited KD exhibits very strong transverse anisotropy (*g*_xx_ = 0.641, *g*_yy_ = 1.288, and *g*_zz_ = 8.531), leading to relaxation from this state with an estimated theoretical barrier for magnetization reversal (*U*_cal_) of 800.4 cm^−1^ (Fig. 1(c)). In contrast, the g tensors of DyScS(C_8_H_6_)_2_ remain Ising-like up to the third excited state. Here, relaxation also occurs from the third excited state, but with a significantly lower energy barrier of 520.3 cm^−1^ (Fig. S2†). This difference may be attributed to the shorter Dy–S bond distance in the cage structures, highlighting the crucial role of the cage in enhancing performance. Here it is noteworthy that the magnetic relaxation diagram computed using this approach is derived from transition matrix elements of the magnetic moment between opposite magnetization states (*n*+ → *n*−) provides and it provides only a qualitative picture of the magnetic relaxation.

### Spin phonon relaxation in DyScS@C_82_

**Fig. 2:**
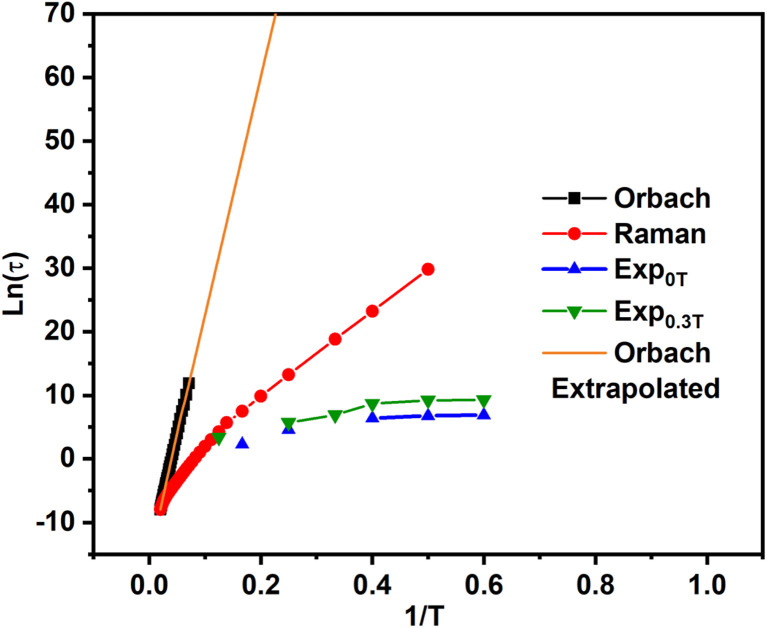
The relaxation times from the Orbach and Raman processes in comparison to the experimental relaxation times.

For first-order transitions, the rate at which spin states |*a*〉 and |*b*〉 change is given by an eqn (5), first and second terms represent spin transitions caused by phonon absorption and emission, respectively. In perfectly axial Kramer systems without external time-reversal symmetry breaking interactions, direct *J*_z_ = 15/2 → *J*_z_ = −15/2 transitions are prohibited. Relaxation based on the given equation must occur through an excited state *via* phonon absorption. Whereas, second-order transitions involve two phonons simultaneously facilitating spin relaxation through an intermediate spin state |*c*〉 within the *J* = 15/2 manifold. The population transfer rate between spin states |*a*〉 and |*b*〉 in this case is described by another eqn (8). Here, *G*^2−Ph^ accounts for energy conservation and phonon thermal population in two-phonon interactions, similar to *G*^1−Ph^ for single-phonon processes. This process is mediated by the excited spin state |*c*〉. The spin-relaxation time is calculated using the lattice force constants, spin-phonon coupling coefficients (∂*Ĥ*_s_/∂*Q*_α_), in eqn (4) and (6), as mentioned earlier. Fig. 2 displays results from both first- and second-order perturbation theory, alongside the best fit to experimental data. First-order theory shows the expected exponential relationship between spin relaxation and temperature (*T*). Raman relaxation, however, the effective reversal barrier *U*_eff_ and the pre-exponential factor (*τ*_0_) were determined by fitting the simulated Orbach data to the Arrhenius expression9
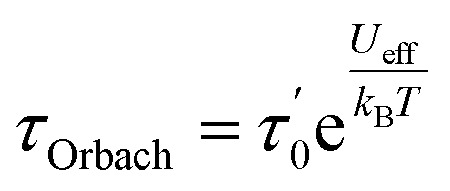
In contrast, the results for Raman relaxation adhere to a more complex mathematical relationship. Recent literature has suggested that Raman relaxation is expected to follow a specific temperature-dependent law.10
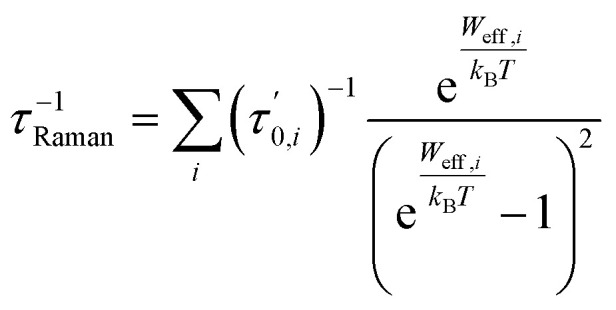


But if there is only a single pair of phonons are involved, where one phonon excites the system and another facilitates relaxation, the Raman relaxations are also simplified to11
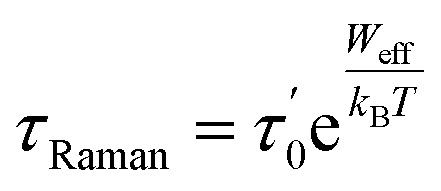


Based on Redfield equations, spin-phonon coupling coefficients are utilised to compute the spin-phonon relaxation time. One- and two-phonon processes have been simulated using second- and fourth-order density matrix time-dependent perturbation theory.^8,69,73^ These simulations are conducted using the open-source programme MolForge, which can be downloaded from github.com/LunghiGroup/MolForge.

High temperatures see the dominance of the Orbach process, whereas the Raman process prevails at low temperatures. In Fig. 2, by plotting ln(*τ*) *vs.* 1/*T*, the slope of the curve at high temperatures gives the *U*_eff_ value of 264 cm^−1^, which also coincides with the energy of the first excited Kramers Doublet (275.2 cm^−1^). The *U*_cal_ value obtained from CASSCF calculations using the Molcas suite differs from this result because the previous method does not account for the effects of spin-phonon relaxation in its calculations. Although not identical, the similar molecule Dy_2_S@C_82_ studied by Chen, Krylov *et al.*^74,75^ exhibits exchange-coupled behaviour at extremely low temperatures. However, at moderate and high temperatures, it demonstrates single-ion properties, making it suitable for comparison. Notably, it shows a similar high-temperature *U*_eff_ value of 363 cm^−1^, which can be compared to the *U*_eff_ of 264 cm^−1^ obtained using our method.

This aligns with the definition of the Orbach relaxation mechanism, which stipulates that phonons must be in resonance with the electronic states. However, the Orbach curve does not remain linear at low temperatures, indicating that other relaxation mechanisms, such as Raman relaxation, may become relevant. This is understandable because, at high temperatures, higher excited states are occupied, and phonons resonating with these states lead to relaxation. On the other hand, at low temperature a curve between ln(*τ*) *vs.* 1/*T* for Raman relaxation, the *W*_eff_ value comes out to be 49 cm^−1^, which coincides with the first optical mode (45.7 cm^−1^) at the Γ-point. Recent literature supports the idea that *W*_eff_ should align with the lowest-energy phonons strongly coupled to the magnetic moment.^8,23,67,76,77^ A similar phenomenon has been observed in metallofullerenes in the literature as well.^23,77^ In previous papers, some of us observed that this phonon typically corresponds to one of the lowest energy modes.^11^ This value is also comparable to the *U*_eff_ of 43 cm^−1^ observed for Dy_2_S@C_82_ at moderate temperatures^74,75^ and pointing to an interpreation of the latter as arising from Raman relaxation instead of Orbach as often assumed for these small barriers.^11^ Importantly, our simulations show that for this compound, the crossover from Orbach to Raman relaxation happens at very high temperature and that Raman relaxation completely dominates the spin dynamics.

We note that the calculated relaxation times differ from the experimental results, except at high temperatures, as shown in Fig. 2. The use of the gas-phase approximation might partially explain the deviations between simulations and experiments at low temperature. Another important factor to consider is the omission of QTM contributions in our approach. Even though experiments have been conducted in the presence of a magnetic field, QTM might have not been completely quenched, making comparison to spin-phonon simulations not straightforward. Ideally a systematic experimental study as function of magnetic dilution could provide a more robust comparison. Despite these deviations at low temperature, experiments and simulations start converging at the highest temperatures at which experiments are available, pointing to intra-molecular contributions as the dominant in this regime.

**Fig. 3:**
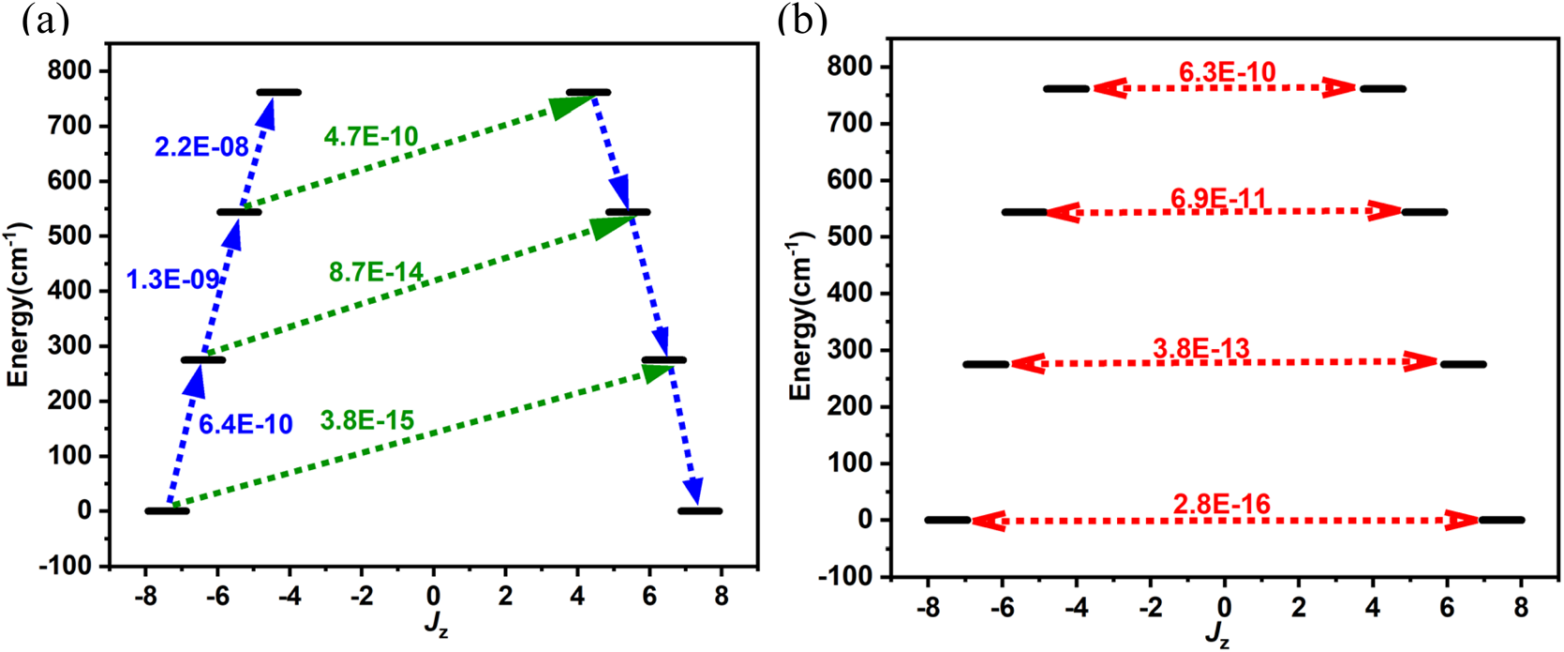
(a) Orbach transition rates computed at 20 K and (b) Raman transition rates computed at 6 K. The Orbach transition rates show the matrix elements of, *Ŵ*^1−ph^ whereas the Raman relaxations show the matrix elements of *Ŵ*^2−ph^. Rates are expressed in ps^−1^ and only rates larger than 10^−16^ are reported explicitly. The *x*-axis displays the computed average magnetic moment for the first four KDs and their energy separation from the ground state.

To gain deeper insights into the relaxation mechanisms of the DyScS@C_82_ complex, we measured transition probabilities for Orbach and the Raman relaxation, as shown in Fig. 3. Fig. 3(a) presents computed transition rates for the Orbach mechanism at 20 K, while Fig. 3(b) illustrates Raman relaxation probabilities at 6 K. It is noteworthy that these values represent the true *ab initio* computed spin-phonon transition rates used to calculate relaxation times, rather than the typical expected dipole moment values associated with such mechanisms. These diagrams suggest that Orbach mechanism is promoted through the absorption of a phonon resonant with the first excited KD, while Raman relaxation is instead described by an intra-KD transition promoted by the simultaneous absorption and emission of pairs of degenerate phonons.

### Examination of vibrational density of states, spin-phonon coupling constants and the molecular vibrations

**Fig. 4:**
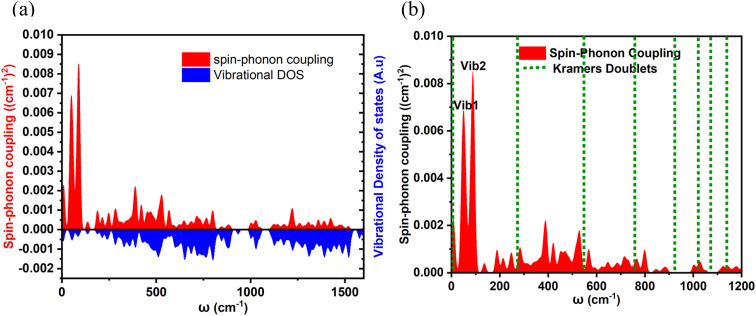
(a) Spin-phonon coupling distribution and the vibrational density of states as functions of energy. A Gaussian smearing with *σ* = 1 cm^−1^ and *σ* = 10 cm^−1^ has been applied to the two functions, respectively. (b) Close-up of the spin-phonon coupling distribution overlapped with the energies of the KDs.

**Fig. 5:**
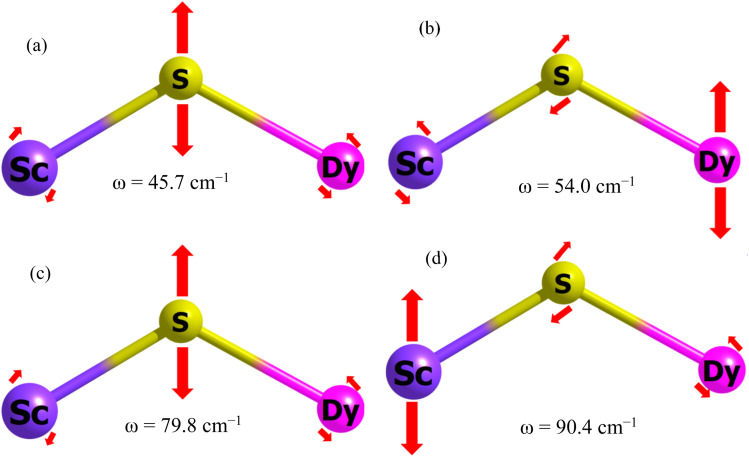
(a) First vibration of **vib1**, (b) second vibration of **vib1** (c) first vibration of **vib2** (d) second vibration of **vib2**. The red arrows indicate the movement, with the size of the arrow representing the extent of motion for that particular atom.

After establishing the temperature dependence of the relaxation times, we focused on the spin-phonon coupling constants and the vibrational density of states (DOS) to decipher the origin of the Raman relaxation observed in the aforementioned section. Fig. 4(a) illustrates the spin-phonon coupling coefficients as a function of frequencies along with the vibrational DOS. Fig. 4(b) presents the detailed spin-phonon coupling coefficients alongside the energies of the eight Kramers doublets (KDs). We found no direct correlation between the vibrational DOS and spin-phonon coupling coefficients at low frequencies, though a partial correlation is evident at higher frequencies. Two regions exhibit very strong spin-phonon coupling: one near 50 cm^−1^ (**vib1**, shown in Fig. 5(a and b)) and another at 87 cm^−1^ (**vib2**, shown in Fig. 5(c and d)). Both modes occur at significantly lower frequencies than the first excited KD, indicating no possibility of Orbach relaxation for this molecule. Closer inspection of these modes revealed that they involve the movement of the Dy–S–Sc moiety inside the cage. Other spin-phonon coupling coefficients at higher frequencies are much smaller.

As expected, since anisotropy should be primarily affected by the first coordination sphere, the highest spin-phonon coupling is observed with the movements of S, Sc, and Dy atoms inside the fullerene. We will now discuss **vib1** and **vib2** in detail. Both modes are labelled in Fig. 4(a) and are pictorially represented in Fig. 5. These modes were selected based on their highest spin-phonon coupling coefficients and exhibit similar types of motions. **vib1** consists of the first two phonons at 45.7 cm^−1^ and 54.0 cm^−1^, involving two different vibrations. This aligns with our *W*_eff_ value (49 cm^−1^), as well as the low temperature energy barrier of the Dy_2_S@C_82_. First is a wagging-like vibration, where the Dy–S−Sc angle (and Sc–S, Dy–S bond distances) changes from 119.8° (2.331 Å, 2.514 Å) to 115.8° (2.395 Å, 2.550 Å). The second vibration is more like a scissoring motion, with Dy^III^ moving more, changing the angle from 115.4° (2.333 Å, 2.532 Å) to 124.4° (2.328 Å, 2.518 Å). The **vib2** consists of two phonons at 79.8 cm^−1^ and 90.4 cm^−1^. The first phonon corresponds to a similar wagging mode as in **vib1**, where the bond angle and bond distances change from 117.7° (2.357 Å, 2.536 Å) to 119.8° (2.330 Å, 2.514 Å). The second phonon in **vib2** corresponds to a scissoring-like bending motion, with Sc^III^ moving more, changing the angle from 117.1° (2.347 Å, 2.509 Å) to 122.7° (2.335 Å, 2.524 Å).

Additionally, it was observed that cage atoms begin to visibly vibrate only at the eighth phonon mode, which occurs at 210.9 cm^−1^. This corresponds to the previously discussed *U*_eff_ value (264 cm^−1^) and the one reported for Dy_2_S@C_82_ in the context of Orbach relaxation. This suggests that the cage indeed provides a largely rigid ligand environment that does not contribute to Raman relaxation.

## Discussion and conclusions

The field of molecular magnetism has made significant progress over the past three decades, with the discovery of compounds exhibiting remarkably *U*_eff_ values and magnetic hysteresis at increasingly high temperatures. However, the field now faces a critical juncture, as traditional strategies for increasing the effective energy barrier in single-ion complexes may be approaching their practical limits. Future advancements in designing molecular compounds with extended spin lifetimes will likely stem from a more comprehensive understanding of the entire spin relaxation process. This necessitates a shift in focus beyond the singular pursuit of higher *U*_eff_ values, encompassing a broader analysis of relaxation phenomena, including the often overlooked preexponential factor *τ*_0_. On the other hand, first-principles simulations have recently demonstrated their capacity to accurately model spin relaxation processes, enabling the estimation of spin relaxation times without necessarily relying on experimental data and providing an unbiased means to benchmark our understanding of spin-phonon interactions and relaxation mechanisms. This computational approach enables researchers to directly interpret relaxation experiments without relying on phenomenological models. By providing a more fundamental and system-specific understanding, these simulations offer a powerful alternative to traditional interpretative frameworks, potentially leading to more accurate and insightful analyses of spin relaxation phenomena in molecular magnetic materials.

In this paper, we have delved into the spin-phonon relaxation mechanisms within fullerene molecules, focusing on the high-performance SMM DyScS@C_82_, which shows a *T*_B_ of 7.3 K. The study of spin-phonon relaxation in DyScS@C_82_ SMMs offers significant insights into how the interactions between molecular vibrations and electronic spin states influence the magnetic behaviour of these systems. The research highlights the importance of a molecular-level understanding of blocking temperatures in SMMs, which has long been a critical challenge in advancing practical applications of these molecules. The key-findings of this work are outlined below.

### (i) Role of cage in SMM performance

The fullerene cage plays a crucial role in stabilizing the molecular fragment and modifying its geometry to enhance SMM performance. The spatial constraints imposed by the cage shorten Dy-C_cage_ and Dy–S bond distances, which, while not enhancing axiality through the ligand field, lead to a higher effective energy barrier (*U*_cal_). This effect is evident in DyScS@C_82_ (800.4 cm^−1^) compared to DyScS(C_8_H_6_)_2_ (520.3 cm^−1^), demonstrating that the cage-induced bond contraction contributes significantly to improved SMM behavior.

### (ii) Mode of relaxation

Analysis of relaxation time *versus* temperature graphs reveals that the predominant relaxation mechanism is the Raman process. The Orbach mechanism involves phonon absorption resonant with the first excited KD, while Raman relaxation occurs *via* intra-KD transitions through simultaneous absorption and emission of degenerate phonons. The highest spin-phonon coupling coefficients occur at low frequencies, significantly lower than the energy of the first Kramers doublet (KD), suggesting that the relaxation mechanism cannot be attributed to the Orbach process.

### (iii) Spin-phonon coupling dynamics in DyScS@C_82_

At lower frequencies, we observed a pronounced phenomenon of spin-phonon coupling, where interactions between spin states and vibrational modes are notably strong. The lowest energy optical phonons arise from specific motions within the {Dy–S−Sc} moiety, exhibiting a robust coupling with the spin states of the system. It is noteworthy that vibrations originating from the fullerene cage occur at higher frequencies, beginning prominently at 210.9 cm^−1^. These vibrations primarily represent the structural dynamics of the cage itself, with minimal interaction with the enclosed Dy–S−Sc moiety, thereby not leading to significant spin-phonon coupling. Thus, the cage clearly offers a significant advantage and does not contribute to spin-phonon relaxation.

### (iv) How cluster atom vibrations drive relaxation

We have identified four distinct low-lying phonons crucial to the relaxation dynamics of the system. These phonons involve specific bending motions that cause variations in the Dy–S–Sc bond angles, thereby initiating relaxation processes. The changes in Dy–S–Sc bond angles are known to influence the axial ligand field: an increase in bond angle reduces the axial ligand field strength, consequently promoting transverse anisotropy and facilitating relaxation pathways within the system. Unfortunately, these phonons predominantly operate at low frequencies, which poses a limitation on the system's ability to achieve high blocking temperatures. This characteristic underscores the importance of understanding and managing these low-frequency vibrational modes in the design and engineering of materials aimed at enhancing thermal stability and magnetic properties.

Our study reveals that while the fullerene cage improves stability and crystal field splitting of the {DyScS} fragment, it does not completely eliminate low-frequency vibrational modes within the cluster, which contribute to spin-phonon relaxation. These key low-lying phonons affect Dy–S–Sc bond angles, leading to transverse anisotropy and relaxation pathways. For enhanced performance in future EMFs, designs should focus on stronger Dy-ligand bonds, smaller cage sizes to reduce internal tumbling, and maintaining the cluster close to its equilibrium geometry. This is exemplified in the literature evidences for instance, DyLu_2_N@I_h_(7)-C_80_, with a blocking temperature of 9 K,^30^ demonstrates stronger Dy–ligand interaction, while optimal combinations like in Dy@Cs(6)-C_81_N,^26^ show fewer low-energy phonons coupled to spin states, leading to even higher blocking temperatures. This novel approach not only clarifies the spin-phonon mechanism for EMF-based SMMs but also provides a strategic pathway to enhance the performance of future EMF-based SMMs. Overall, this study advances the understanding of spin-phonon relaxation in SMMs and provides valuable guidelines for future molecular design aimed at increasing *T*_B_ values. By focusing on the interplay between molecular structure, vibrational modes, and spin dynamics, it opens up new avenues for the development of high-performance SMMs, particularly in the context of fullerene-based systems where rigid molecular environments can stabilize low-coordinate lanthanide centers.

## Author contributions

GR and AL designed and supervised the investigations; TS carried out DFT, periodic DFT, CASSCF, and ab initio spin-phonon relaxation calculations; RKT and SD contributed to the CASSCF calculations; LAM assisted in troubleshooting the *ab initio* spin-phonon relaxation calculations.

## Conflicts of interest

There are no conflicts of interest to declare.

## Supplementary Material

SC-016-D4SC07786E-s001

## Data Availability

The data supporting this article have been included as part of the ESI.†
