# Supplementary material for: The mechanism of spin-phonon relaxation in endohedral metallofullerene single molecule magnets
Source: Chem Sci. 2025 Jun 9;16(28):13012–21. doi: 10.1039/d4sc07786e (PMC12175316; doi:10.1039/d4sc07786e)
Supplement: SC-016-D4SC07786E-s001 [file SC-016-D4SC07786E-s001.pdf]

Supplementary Information (SI) for Chemical Science.
This journal is © The Royal Society of Chemistry 2025

The Mechanism of Spin-Phonon Relaxation in Endohedral Metallofullerene Single Molecule Magnets

Tanu Sharma[a], Rupesh Kumar Tiwari[a], Sourav Dey[a], Lorenzo A. Mariano[b], Alessandro Lunghi[b]* and Gopalan Rajaraman[a]*

a- Department of Chemistry, Indian Institute of Technology Bombay, Mumbai, Maharashtra, 400076, India. Email: rajaraman@chem.iitb.ac.in.

b- School of Physics and AMBER Research Centre, Trinity College, Dublin 2, Ireland. Email: lunghia@tcd.ie

**Table S1**: Summary of all the Endohedral metallofullerene based SMMs along with blocking temperature and the U_{eff}.						
Sr. No.	Molecule	$T_{B,100s}$ (K)	$T_{B,hys}$ (K)(Field sweep mTs−1)is given in bracket)	$T_{B,ZFC}$ (K)(temperature sweep (K min-1) used is given in bracket)	U_{eff} (K)	Ref
1	$DySc_2N@D_3(6140)-C_{68}$	2.3	5 (2.9)	3.8 (5)	23.6	[1]
2	$DySc_2N@D_{5h}(6)-C_{80}$	3.6	7 (2.9)	5.9 (5)	17.7	[1]
3	$DySc_2N@I_h(7)-C_{80}$	4.6	7 (2.9)	6.9 (5)	23.6	[2]
4	$DyY_2N@I_h(7)-C_{80}$	~6	8 (2.9)	8.4 (5)	929	[3]
5	$DyLu_2N@I_h(7)-C_{80}$	~6.5	9 (2.9)	9.5 (5)	24.2	[3, 4]
6	HoSc2N@Ih(7)-C_{80}	—	—	—	16.5	[5]
7	$Dy_2ScN@I_h(7)-C_{80}$	5	7 (2.9)	8 (5)	10.7/1735	[6]
8	$Dy_2YN@I_h(7)-C_{80}$	~3.5	5 (2.9)	4.7 (5)	43.8/680	[3]
9	$Dy_2LaN@I_h(7)-C_{80}$	~2	4 (2.9)	3.3 (5)	—	[3]
10	$Dy_2LuN@I_h(7)-C_{80}$	5.2	8 (2.9)	8 (5)	4.3	[3, 4]
11	$Dy_2GdN@I_h(7)-C_{80}$	~1.5	~1.8 (5.3)	—	15.1	[7]
12	$DyErScN@I_h(7)-C_{80}$	~4.5	9 (33)	~8 (3)	12.5	[8]
13	$Tb_2ScN@I_h(7)-C_{80}$	0.4	~0.4 (3.3)	—	1/10.5/56.4	[9]
14	$Dy_2ScN@D_{5h}(6)-C_{80}$	2.6	7 (2.9)	5.3 (5)	8.4	[1]
15	$Dy_2ScN@D_s(51365)-C_{84}$	~1.8	5 (2.9)	3.3 (5)	—	[1]
16	$Dy_3N@I_h(7)-C_{80}$	—	~2 (0.8)	—	—	[10]
17	TbCN@C2v(19138)-C76b	—	—	—	12	[11]
18	TbCN@C2(5)-C82b	—	—	—	10–20	[12]
19	TbCN@Cs(6)-C82b	—	—	—	10–20	[12]
20	TbCN@C2v(9)-C82b	—	—	—	10–20	[12]
21	$Dy_2O@C_s(10528)-C_{72}$	3.4	7 (2.9)	8 (5)	—	[13]
22	$Dy_2O@C_2(13333)-C_{74}$	5.0	14 (2.9)	14 (5)	—	[13]
23	$Dy_2O@C_{2v}(5)-C_{80}$	3.2	6 (2.9)	11 (5)	25.9	[14]
24	$Dy_2O@C_s(6)-C_{82}$	2.8	6 (2.9)	10 (5)	10.8	[15]
25	$Dy_2O@C_{3v}(8)-C_{82}$	5.9	7 (2.9)	9 (5)	7.8	[15]
26	$Dy_2O@C_{2v}(9)-C_{82}$	3.7	7 (2.9)	8 (5)	18.6	[15]
27	$Dy_2O@C_1(26)-C_{88}$	6	8 (2.9)	10.5 (5)	20.4	[16]
28	$Dy_2O@C_s(32)-C_{88}$	4.6	8 (2.9)	8.5 (5)	—	[16]
29	$Dy_2O@D_2(35)-C_{88}$	3.9	8 (2.9)	8.5 (5)	—	[16]
30	$Dy_2S@C_s(10528)-C_{72}$	—	3.0 (8.33)	—	—	[17]
31	$Dy_2S@C_s(6)-C_{82}$	—	3.0 (8.33)	—	17.8	[17, 18]

32	$Dy_2S@C_{3v}(8)\text{-}C_{82}$	2	5 (8.33)	4.0 (5)	6	[17, 18]
33	$DyScS@C_s(6)\text{-}C_{82}$	~4	9 (10)	7.3 (5)	15.2	[19]
34	$DyScS@C_{3v}(8)\text{-}C_{82}$	~2	9 (10)	7.3 (5)	6.5	[19]
35	$DyYTiC@I_h(7)\text{-}C_{80}$	~5	7 (2.9)	8 (5)	14.9	[20]
36	$Dy_2TiC@I_h(7)\text{-}C_{80}$	1.7	3 (5)	—	9.5	[21]
37	$Dy_2TiC_2@I_h(7)\text{-}C_{80}$	—	1.8 (5)	—	—	[21]
38	$Dy_2TiC@D_{5h}(6)\text{-}C_{80}$	—	1.8 (5)	—	—	[21]
39	$Dy_2C_2@C_s(6)\text{-}C_{82}$	—	3.0 (8.33)	—	17.4	[17]
40	$Dy_2C_2@C_s(32)\text{-}C_{88}$	—	2.1 (2.9)	—	—	[16]
41	$Dy_2C_2@D_2(35)\text{-}C_{88}$	—	2.1 (2.9)			[16]
42	$Dy@C_s(6)\text{-}C_{81}N$	45	39 (3.5)/60 (10)	69 (1)	—	[22]
43	$Dy_2@C_{80}(CH_2Ph)$	18	22 (2.9)	21.9 (5)	613	[23]
44	$Dy_2@C_{79}N$	12	24 (20)	21 (3)	669	[24]
45	$Tb_2@C_{79}N$	24	26 (2.9)	28 (5)	757	[25]
46	Tb2@C80(CH2Ph)	25.2	27 (9.5)	28.9 (5)	799	[26]
47	$Tb_2@C_{80}(CF_3)$	25	26 (2.9)	28.5 (5)	801	[27]
48	$Ho_2@C_{80}(CH_2Ph)$	—	—	—	334	[26]
49	Er2@C80(CH2Ph)a	—	—	—	—	[26]
50	$TbGd@C_{80}(CH_2Ph)$	—	—	14.4 (5)	—	[26]
51	$TbY@C_{80}(CH_2Ph)$	—	5 (2.9)	5 (5)	—	[26]
52	Nd2@C80(CF3)a	—	—	—	—	[28]
53	Gd2@C79Na	—	—	—	6.5	[29, 30]
54	$DyEr@C_{3v}(8)\text{-}C_{82}$	—	3 (33)	5 (3)		[31]

Table S2: |V(r)/G(r)| ratio of $DySSc@C_{82}$ and Fragment $DyScS(C_8H_6)_2$. (Here V(r) is the Virial Field function, G(r) is the electronic kinetic energy density, |V(r)/G(r)| is the ratio of Virial field function to Electronic kinetic energy.

Bond	V(r)	G(r)	$(\nabla^2\rho(r)$	H(r)	|V(r)/G(r)|
$DySSc@C_{82}$					
Dy-S	−0.071	0.056	0.159	−0.015	1.285
Dy-C	−0.074	0.062	0.202	−0.008	1.189
Dy-S	−0.081	0.043	0.137	−0.011	1.143
Dy-C	−0.076	0.062	0.194	−0.014	1.219

Note: |V(r)/G(r)| < 1 – ionic interaction, |V(r)/G(r)| > 2 – covalent interaction, 1<|V(r)/G(r)<2 – intermediate interaction.

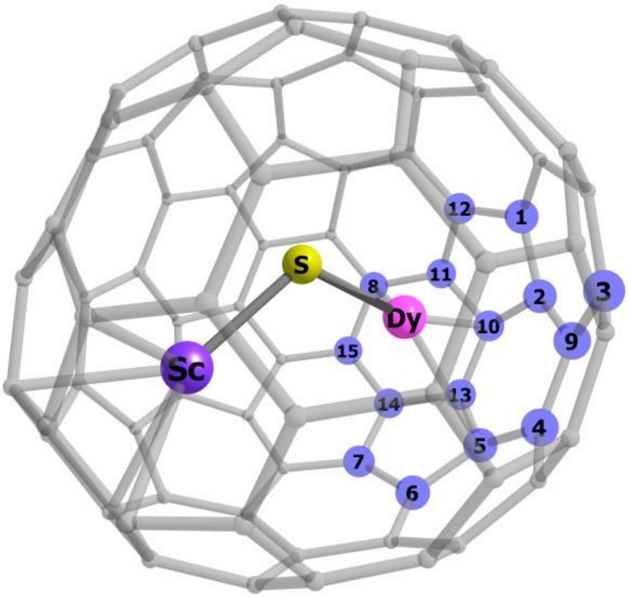

Figure S1:DyScS@C_{82} structure showing the marked C atoms as the one having considerable delocalization indices.

Table S3: Delocalisation index δ(Dy, C_{cage}) between Dy^{III} and the cage carbon atoms.

Carbon number	δ(Dy, C_{cage})
1	0.236
2	0.315
3	0.101
4	0.208
5	0.276
6	0.163
7	0.163
8	0.237
9	0.237
10	0.345
11	0.315
12	0.236
13	0.328
14	0.276
15	0.208

**Table S4**: Energies, g-tensor, angle of the ground excited g_{zz} with the ground g_{zz} in DyScS@C_{82} molecule and the DyScS(C_8H_6).					
KD	Energies (cm^{-1})	g_{xx}	g_{yy}	g_{zz}	Angle (°)
DyScS@C_{82}					
1	0.0	0.002	0.003	19.925	
2	239.3	0.009	0.012	17.078	2.5
3	471.0	0.190	0.235	14.133	5.9
4	659.7	0.249	0.567	11.697	20.1
5	800.4	0.641	1.288	8.531	4.4
6	889.7	6.186	5.695	3.801	45.7
7	941.6	0.996	3.247	14.668	96.1
8	1011.6	0.182	1.136	18.890	89.8
DyScS(C_8H_6)$_2$					
1	0.0	0.001	0.001	20.032	
2	271.9	0.012	0.018	17.726	27.8
3	389.9	0.058	0.079	14.218	21.5
4	520.3	0.875	1.029	12.453	16.6
5	596.8	1.725	3.809	10.140	36.5
6	671.5	3.383	5.859	9.881	86.8
7	764.8	0.909	1.938	16.382	87.9
8	988.9	0.043	0.075	20.059	93.4

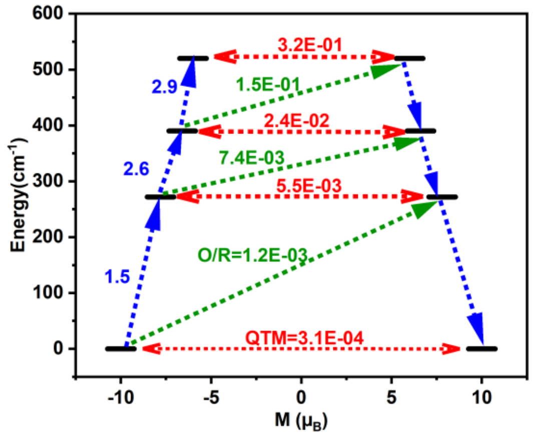

Figure S2: Relaxation mechanism of spin reversal in fragment DyScS(C_8H_6)$_2$

Geometry optimisation input file

&GLOBAL

PROJECT

PRINT_LEVEL MEDIUM

```
  RUN_TYPE     GEO_OPT
  FLUSH_SHOULD_FLUSH  T
&END GLOBAL
&MOTION
  &GEO_OPT
    TYPE MINIMIZATION
    MAX_FORCE 1.0E-07
    MAX_ITER 1000
    OPTIMIZER  BFGS
  &END GEO_OPT
      &CONSTRAINT
    &FIXED_ATOMS
    &END FIXED_ATOMS
   &END CONSTRAINT
&END MOTION
&FORCE_EVAL
   METHOD Quickstep
  &DFT
    BASIS_SET_FILE_NAME  /opt/cp2k-2.4.0/cp2k-2.4.0/tests/QS/BASIS_MOLOPT
    POTENTIAL_FILE_NAME  /opt/cp2k-2.4.0/cp2k-2.4.0/tests/QS/GTH_POTENTIALS
    UKS T
    CHARGE  0
    MULTIPLICITY 1
    &MGRID
      NGRIDS 5
      CUTOFF 1000
      REL_CUTOFF 60
    &END MGRID
    &QS
    EXTRAPOLATION PS
```

```
EXTRAPOLATION_ORDER 4
EPS_DEFAULT 1.0E-6
&END QS
  &POISSON                !Necessary to define POISSON section in non-perdiodic
     PERIODIC NONE        !boudary conditions as the default assumes PBCs
     POISSON_SOLVER MT
  &END POISSON
&SCF
  SCF_GUESS    ATOMIC
  EPS_SCF      1.0E-8
  MAX_SCF      200
  &MIXING  T
    METHOD BROYDEN_MIXING
    ALPHA        0.1
    BETA         1.5
    NBROYDEN      8
  &END MIXING
  &OUTER_SCF
    EPS_SCF                    1.0E-8
    MAX_SCF                    100
    OPTIMIZER                  DIIS
  &END OUTER_SCF
  &OT ON
    PRECONDITIONER             FULL_KINETIC
    MINIMIZER                  CG
    N_HISTORY_VEC              7
    ENERGY_GAP     1.0000000000000000E-04
  &END OT
   &PRINT
    &RESTART
```

```
        LOG_PRINT_KEY             T
       &END RESTART
     &END PRINT
   &END SCF
    &XC
     &XC_GRID
      XC_SMOOTH_RHO             NN50
      XC_DERIV                  NN50_SMOOTH
     &END XC_GRID
     &XC_FUNCTIONAL             PBE
     &END XC_FUNCTIONAL
      &VDW_POTENTIAL
        POTENTIAL_TYPE  PAIR_POTENTIAL
        &PAIR_POTENTIAL
          TYPE  DFTD3
           REFERENCE_FUNCTIONAL PBE
           PARAMETER_FILE_NAME ./dftd3.dat
           R_CUTOFF 10
        &END PAIR_POTENTIAL
      &END VDW_POTENTIAL
    DENSITY_CUTOFF   1.0E-9
    GRADIENT_CUTOFF  1.0E-9
    TAU_CUTOFF       1.0E-9
   &END XC
&END DFT
 &SUBSYS
   &TOPOLOGY
    &CENTER_COORDINATES     T
    &END CENTER_COORDINATES
   &END TOPOLOGY
```

```
    &PRINT
    &END PRINT
    &CELL
      A     20.00000000      0.000000000    0.000000000
      B     0.000000000      20.00000000    0.000000000
      C     0.000000000      0.000000000    20.00000000
    PERIODIC NONE
    &END CELL
&COORD
C        9.852943614     11.496206211     13.760374766
C       11.078699185     12.098340519     13.323924624
C       12.098293098     11.078758088     13.323927748
C       11.496171073      9.852997063     13.760379490
C       10.110906752     10.110945522     14.032292840
C       11.919856109      8.595599571     13.233905836
C       13.021171913      8.587288375     12.334566679
C       13.634633003      9.817630026     11.907139897
C       13.130627151     11.067957675     12.346097621
C       13.165905149     12.153293467     11.401093405
C       13.674682202     12.019634709     10.049549163
C        7.945408693     13.488488475      8.859082876
C       14.055436188      9.646597357     10.536933061
C       13.767743439      8.280243283     10.173966009
C       13.078825340      7.640548966     11.247822717
C       13.488488735      7.945470100      8.859099399
C       13.448421126      8.968290064      7.848120805
C       13.664563895     10.329365470      8.185970982
C       12.954579357     11.311396959      7.397700356
C       11.888183661     10.884173251      6.514327762
C       11.573070437      9.513322781      6.276373389
```

C	12.437856751	8.568631013	6.881787049
C	11.930410215	7.297375241	7.298504347
C	12.481873482	6.956867545	8.581187669
C	11.676013768	6.393471531	9.611382199
C	12.011434023	6.723534943	10.991202694
C	10.992759516	6.654215328	11.977407730
C	10.942880747	7.572684970	13.077753275
C	9.645062702	6.301828540	11.635584910
C	7.075882756	7.075878655	10.784104583
C	10.297841568	6.013479554	9.270470241
C	9.844925473	6.193479766	7.920557801
C	10.642420415	6.922301753	6.971099568
C	9.793153536	7.805527951	6.218755969
C	10.188892887	9.153967315	5.969989327
C	8.432909750	7.438555941	6.540292360
C	8.453140635	6.417191993	7.570204450
C	7.448639289	6.401829328	8.578932837
C	7.899092186	6.246518570	9.938372801
C	7.494615346	7.494619454	12.072337033
C	6.246515239	7.899075853	9.938367839
C	6.401834602	7.448620509	8.578929559
C	6.417188880	8.453118653	7.570197620
C	6.193461880	9.844902351	7.920547231
C	6.013452631	10.297820721	9.270457722
C	5.983224170	9.280827107	10.312704303
C	7.438557086	8.432896602	6.540288934
C	7.805515759	9.793143450	6.218750704
C	9.153951635	10.188896982	5.969986574
C	6.922278077	10.642402862	6.971088958
C	7.297335860	11.930397934	7.298490717

C	8.568587378	12.437857091	6.881775779
C	9.513290689	11.573079314	6.276367654
C	10.884136998	11.888208521	6.514324978
C	7.640485199	13.078828449	11.247806582
C	9.280845084	5.983243445	10.312713673
C	10.685656929	14.011078681	9.569667554
C	14.011047862	10.685726315	9.569677388
C	13.021733332	13.021790041	9.221007237
C	12.019567402	13.674728850	10.049544076
C	12.153228224	13.165958278	11.401090232
C	11.067890034	13.130671366	12.346091352
C	9.817558106	13.634661917	11.907128463
C	9.646524741	14.055459114	10.536919854
C	8.587222123	13.021188662	12.334553536
C	8.280174905	13.767750764	10.173949628
C	12.639977845	12.640026385	7.878912209
C	6.723483706	12.011426467	10.991187014
C	6.393428313	11.675998316	9.611366511
C	6.956818062	12.481861294	8.581171188
C	6.654172549	10.992754014	11.977394995
C	7.572639293	10.942888565	13.077743662
C	8.595542964	11.919875461	13.233895924
C	7.818565287	9.556004225	13.393229941
C	7.020441342	8.755758767	12.528986646
C	6.301801586	9.645052255	11.635575358
C	8.968232185	13.448428866	7.848107527
C	8.755758687	7.020460775	12.528991262
C	9.555992612	7.818596216	13.393235345
C	9.117486870	9.117514073	13.808233119
C	11.311346073	12.954611336	7.397695953

```
C       10.329304198     13.664587099      8.185961290
S        9.692245827      9.692271395     10.364521316
Sc       8.419064249      8.419076518      8.885125934
Y       11.354575819     11.354610591      9.473953315
&END COORD
    &KIND C
      ELEMENT     C
      BASIS_SET    DZVP-MOLOPT-GTH
      POTENTIAL    GTH-PBE-q4
    &END KIND
    &KIND S
      ELEMENT     S
      BASIS_SET    DZVP-MOLOPT-GTH
      POTENTIAL    GTH-PBE-q6
    &END KIND
    &KIND Sc
      ELEMENT     Sc
      BASIS_SET    DZVP-MOLOPT-SR-GTH
      POTENTIAL    GTH-PBE-q11
    &END KIND
    &KIND Y
      ELEMENT     Y
      BASIS_SET    DZVP-MOLOPT-SR-GTH
      POTENTIAL    GTH-PBE-q11
    &END KIND
    &END SUBSYS
  &END FORCE_EVAL
```

References

1. C. Schlesier, L. Spree, A. Kostanyan, R. Westerström, A. Brandenburg, A. U. Wolter, S. Yang, T. Greber and A. A. Popov, *Chemical Communications*, 2018, **54**, 9730-9733.
2. R. Westerström, J. Dreiser, C. Piamonteze, M. Muntwiler, S. Weyeneth, H. Brune, S. Rusponi, F. Nolting, A. Popov and S. Yang, *Journal of the American Chemical Society*, 2012, **134**, 9840-9843.
3. Y. Hao, G. Velkos, S. Schiemenz, M. Rosenkranz, Y. Wang, B. Büchner, S. M. Avdoshenko, A. A. Popov and F. Liu, *Inorganic Chemistry Frontiers*, 2023, **10**, 468-484.
4. L. Spree, C. Schlesier, A. Kostanyan, R. Westerström, T. Greber, B. Büchner, S. M. Avdoshenko and A. A. Popov, *Chemistry–A European Journal*, 2020, **26**, 2436-2449.
5. J. Dreiser, R. Westerström, Y. Zhang, A. A. Popov, L. Dunsch, K. Krämer, S. X. Liu, S. Decurtins and T. Greber, *Chemistry–A European Journal*, 2014, **20**, 13536-13540.
6. D. Krylov, F. Liu, S. Avdoshenko, L. Spree, B. Weise, A. Waske, A. Wolter, B. Büchner and A. Popov, *Chemical Communications*, 2017, **53**, 7901-7904.
7. A. Kostanyan, C. Schlesier, R. Westerström, J. Dreiser, F. Fritz, B. Büchner, A. A. Popov, C. Piamonteze and T. Greber, *Physical Review B*, 2021, **103**, 014404.
8. M. Nie, J. Xiong, C. Zhao, H. Meng, K. Zhang, Y. Han, J. Li, B. Wang, L. Feng and C. Wang, *Nano Research*, 2019, **12**, 1727-1731.
9. A. Kostanyan, R. Westerström, D. Kunhardt, B. Büchner, A. A. Popov and T. Greber, *Physical Review B*, 2020, **101**, 134429.
10. R. Westerström, J. Dreiser, C. Piamonteze, M. Muntwiler, S. Weyeneth, K. Krämer, S.-X. Liu, S. Decurtins, A. Popov and S. Yang, *Physical Review B*, 2014, **89**, 060406.
11. F. Liu, S. Wang, C. L. Gao, Q. Deng, X. Zhu, A. Kostanyan, R. Westerström, F. Jin, S. Y. Xie and A. A. Popov, *Angewandte Chemie*, 2017, **129**, 1856-1860.
12. F. Liu, C.-L. Gao, Q. Deng, X. Zhu, A. Kostanyan, R. Westerström, S. Wang, Y.-Z. Tan, J. Tao and S.-Y. Xie, *Journal of the American Chemical Society*, 2016, **138**, 14764-14771.
13. G. Velkos, W. Yang, Y.-R. Yao, S. M. Sudarkova, X. Liu, B. Büchner, S. M. Avdoshenko, N. Chen and A. A. Popov, *Chemical science*, 2020, **11**, 4766-4772.
14. G. Velkos, W. Yang, Y.-R. Yao, S. M. Sudarkova, F. Liu, S. M. Avdoshenko, N. Chen and A. A. Popov, *Chemical Communications*, 2022, **58**, 7164-7167.
15. W. Yang, G. Velkos, F. Liu, S. M. Sudarkova, Y. Wang, J. Zhuang, H. Zhang, X. Li, X. Zhang and B. Büchner, *Advanced Science*, 2019, **6**, 1901352.
16. W. Yang, G. Velkos, S. Sudarkova, B. Büchner, S. M. Avdoshenko, F. Liu, A. A. Popov and N. Chen, *Inorganic Chemistry Frontiers*, 2022, **9**, 5805-5819.
17. C.-H. Chen, D. S. Krylov, S. M. Avdoshenko, F. Liu, L. Spree, R. Yadav, A. Alvertis, L. Hozoi, K. Nenkov and A. Kostanyan, *Chemical science*, 2017, **8**, 6451-6465.
18. D. Krylov, G. Velkos, C.-H. Chen, B. Büchner, A. Kostanyan, T. Greber, S. M. Avdoshenko and A. A. Popov, *Inorganic chemistry frontiers*, 2020, **7**, 3521-3532.
19. W. Cai, J. D. Bocarsly, A. Gomez, R. J. L. Lee, A. Metta-Magaña, R. Seshadri and L. Echegoyen, *Chemical Science*, 2020, **11**, 13129-13136.
20. A. Brandenburg, D. S. Krylov, A. Beger, A. U. Wolter, B. Büchner and A. A. Popov, *Chemical Communications*, 2018, **54**, 10683-10686.
21. K. Junghans, C. Schlesier, A. Kostanyan, N. A. Samoylova, Q. Deng, M. Rosenkranz, S. Schiemenz, R. Westerström, T. Greber and B. Büchner, *Angewandte Chemie International Edition*, 2015, **54**, 13411-13415.
22. Z. Hu, Y. Wang, A. Ullah, G. M. Gutiérrez-Finol, A. Bedoya-Pinto, P. Gargiani, D. Shi, S. Yang, Z. Shi and A. Gaita-Ariño, *Chem*, 2023, **9**, 3613-3622.

23. F. Liu, D. S. Krylov, L. Spree, S. M. Avdoshenko, N. A. Samoylova, M. Rosenkranz, A. Kostanyan, T. Greber, A. U. Wolter and B. Büchner, *Nature communications*, 2017, **8**, 16098.
24. Y. Wang, J. Xiong, J. Su, Z. Hu, F. Ma, R. Sun, X. Tan, H.-L. Sun, B.-W. Wang and Z. Shi, *Nanoscale*, 2020, **12**, 11130-11135.
25. G. Velkos, D. S. Krylov, K. Kirkpatrick, L. Spree, V. Dubrovin, B. Büchner, S. M. Avdoshenko, V. Bezmelnitsyn, S. Davis and P. Faust, *Angewandte Chemie International Edition*, 2019, **58**, 5891-5896.
26. F. Liu, G. Velkos, D. S. Krylov, L. Spree, M. Zalibera, R. Ray, N. A. Samoylova, C.-H. Chen, M. Rosenkranz and S. Schiemenz, *Nature communications*, 2019, **10**, 571.
27. Y. Wang, G. Velkos, N. J. Israel, M. Rosenkranz, B. Büchner, F. Liu and A. A. Popov, *Journal of the American Chemical Society*, 2021, **143**, 18139-18149.
28. W. Yang, G. Velkos, M. Rosenkranz, S. Schiemenz, F. Liu and A. A. Popov, *Advanced Science*, 2024, **11**, 2305190.
29. Z. Hu, B.-W. Dong, Z. Liu, J.-J. Liu, J. Su, C. Yu, J. Xiong, D.-E. Shi, Y. Wang and B.-W. Wang, *Journal of the American Chemical Society*, 2018, **140**, 1123-1130.
30. G. Velkos, D. Krylov, K. Kirkpatrick, X. Liu, L. Spree, A. Wolter, B. Büchner, H. Dorn and A. Popov, *Chemical Communications*, 2018, **54**, 2902-2905.
31. M. Nie, L. Yang, C. Zhao, H. Meng, L. Feng, P. Jin, C. Wang and T. Wang, *Nanoscale*, 2019, **11**, 18612–18618.
